# Sucrose Synthase Enhances Hull Size and Grain Weight by Regulating Cell Division and Starch Accumulation in Transgenic Rice

**DOI:** 10.3390/ijms20204971

**Published:** 2019-10-09

**Authors:** Chunfen Fan, Guangya Wang, Youmei Wang, Ran Zhang, Yanting Wang, Shengqiu Feng, Keming Luo, Liangcai Peng

**Affiliations:** 1 Chongqing Key Laboratory of Plant Resource Conservation and Germplasm Innovation, Institute of Resources Botany, School of Life Sciences, Southwest University, Chongqing 400715, China; luckyfcf@swu.edu.cn (C.F.);; 2 Biomass & Bioenergy Research Centre, College of Plant Science & Technology, Huazhong Agricultural University, Wuhan 430070, China

**Keywords:** sucrose synthase, grain weight, hull size, cell division, starch accumulation

## Abstract

Grain size and weight are two important determinants of grain yield in rice. Although overexpression of sucrose synthase (*SUS*) genes has led to several improvements on cellulose and starch-based traits in transgenic crops, little is reported about *SUS* enhancement of hull size and grain weight in rice. In this study, we selected transgenic rice plants that overexpressed *OsSUS1-6* genes driven with the maize Ubi promoter. Compared to the controls (wild type and empty vector line), all independent *OsSUS* homozygous transgenic lines exhibited considerably increased grain yield and grain weights. Using the representative *OsSUS3* overexpressed transgenic plants, four independent homozygous lines showed much raised cell numbers for larger hull sizes, consistent with their enhanced primary cell wall cellulose biosynthesis and postponed secondary wall synthesis. Accordingly, the *OsSUS3* transgenic lines contained much larger endosperm volume and higher starch levels than those of the controls in the mature grains, leading to increased brown grain weights by 15–19%. Hence, the results have demonstrated that *OsSUS* overexpression could significantly improve hull size and grain weight by dynamically regulating cell division and starch accumulation in the transgenic rice.

## 1. Introduction

Rice is one of the major staple food crops over the world. As major agronomic traits, grain size and weight are tightly associated with grain yield in rice. In principle, grain length, width and thickness basically determine grain size, while hull size and degree of grain filling are key agronomic traits in determining grain weight [1]. Over the past years, large number of QTLs for rice size and weight have been identified [2], and several major genes are relatedly characterized. For instance, *GS3/GL2/GL3.1/qGL3* genes affect grain length [3,4,5,6], *qSW5/GW5/GSE5/GS2/GS5* determine grain width [7,8,9,10,11], and *GL7/GW8* control both grain length and width [12,13,14]. However, genetic manipulation specific for concurrent improvements of both hull size and grain weight in rice remains to be explored.

In higher plants, sucrose is the major form of photosynthesis to transport from source tissues (leaves) to sink tissues (shoot apex, roots, stems and seeds). Cleavage of sucrose is the first step for utilization of the photo-assimilate in various metabolic pathways. The initial cleavage of sucrose in sink organs is catalyzed by either invertase or sucrose synthase (SUS). Invertase catalyses the irreversible hydrolysis of sucrose to glucose and fructose, while SUS catalyzes the reversible conversion of sucrose and a nucleoside diphosphate into the corresponding nucleoside diphosphate-glucose and fructose [15,16,17,18]. Previous studies have showed that invertase can affect seed development in rice [19] and maize [20,21], but whether SUS enzyme has a similar role in crops remains to be explored. 

SUS enzyme plays an important role in carbon partitioning by providing UDPG and ADPG substrates for the synthesis of wall polysaccharides and starch, respectively [22,23,24,25,26,27]. Although it has been characterized that SUS is involved in structural and storage carbohydrates biosynthesis, growth processes, biomass accumulation and wood density in different plant species [28,29,30,31,32,33,34], much remains unknown about SUS’s impact on seed development in rice. In plants, SUS are coded by multiple genes, and members of them play distinct and diverse roles in development. For instance, among the three genes in maize, *ZmSus1* contributes to seed starch biosynthesis, *ZmSus2* is responsible for endosperm cell wall integrity, and *ZmSus3* may be involved in basal endosperm transfer cell formation [35]. In *Arabidopsis*, *SUS5* and *SUS6*, but not *SUS1*–*4*, are specifically involved in callose formation in the sieve plate [36]. Rice has multiple *OsSUS* genes with distinct and partially overlapping expression patterns, but whether the rice SUS isoforms play different roles in seed development remains to be determined.

In this study, we selected transgenic rice plants that overexpressed each of the six *OsSUS* genes in the background of “Zhonghua11” (ZH11) cultivar. We then observed significantly enhanced grain yield and weight of the transgenic plants in the field experiments. Notably, this study performed a detailed examination of the representative transgenic rice plants that overexpressed *OsSUS3* gene, and attempted to interpret how OsSUSs regulate cell division, cellulose biosynthesis and starch accumulation for enhancements of hull size and grain weight in transgenic rice lines. Thus, this study examined the SUS as an important factor that determines grain size and weight, providing a potential genetic strategy for improved grain yield in rice and beyond.

## 2. Results

### 2.1. Phylogenetic and Expression Analyses of OsSUS Family in Rice 

**Figure 1:**
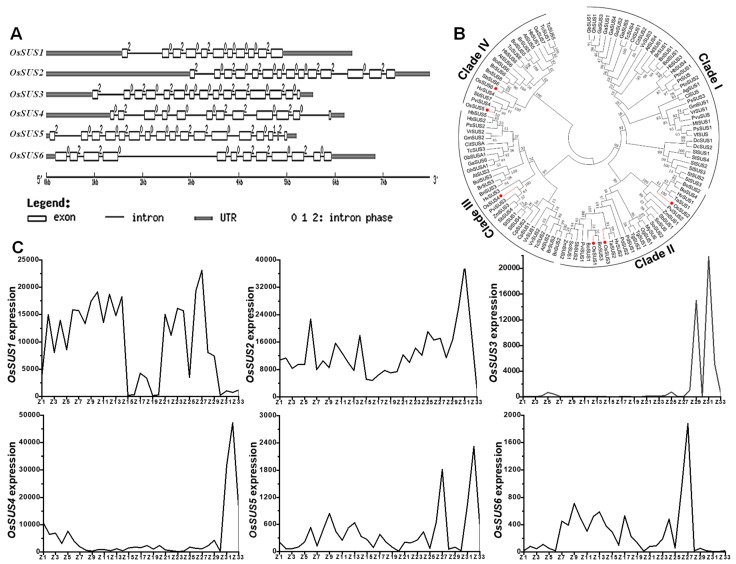
Structurale, phylogenetic and expression analysis of six OsSUS genes. (**A**) Structural comparison of *OsSUS* genes. (**B**) Phylogenetic tree of plant SUS families. (**C**) The expression patterns of *OsSUSs* in ZS97 variety. The x-axis indicates the tissues covering almost all periods of life cycle as shown in Table S2, and the y-axis for the relative gene expression level from the microarray data.

Because there are seven sucrose synthase (SUS) isoforms in rice and *OsSUS7* exhibits 99% similarity with *OsSUS5* [37], this study did not perform any investigation of *OsSUS7*. Among six *OsSUSs* family genes, *OsSUS1*, 4 were examined with 11–12 exons, *OsSUS3*, *4* genes had 14–15 exons, whereas *OsSUS5* and *6* had 17 and 12 exons, respectively (Figure 1A). According to the phylogenetic analysis, the SUS family were classified into four distinct clades in plants: Clade I were of dicot members; Clade II contained monocot members including rice OsSUS1, OsSUS2, and OsSUS3; Clade III and IV consisted of both monocot and dicot members with OsSUS4, OsSUS5, and OsSUS6, respectively (Table S1, Figure 1B). Furthermore, we examined six *OsSUSs* gene expressions based on the microarray analysis (Table S2, Figure 1C). As a result, *OsSUS1* had low expression in leaf and sheath, with high expression in seed imbibition/germination, plumule/radicle, seedling, young shoot/root, panicle, and young stem, whereas *OsSUS2* expressed highly in almost all tissues especially at early endosperm development. By comparison, *OsSUS3* was mainly expressed in the spikelet and endosperm, while *OsSUS4* was specific in endosperm. In addition, *OsSUS5* and *OsSUS6* had a relatively low expression in most tissues. 

### 2.2. Increased Grain Weight in All OsSUSs Transgenic Rice Plants

To examine the *OsSUS’s* roles in rice development, this study generated transgenic rice plants that expressed each *OsSUS* using the maize Ubi promoter of driving gene constitutive expression [38]. A dozen independent, single-locus homozygous *OsSUS1-6* overexpressed transgenic lines (OE, two lines for each *SUS*) were selected for use in this study, based on much increased *OsSUS1-6* transcription levels, compared to the ZH11 (Table S3 and Figure S1).

**Figure 2:**
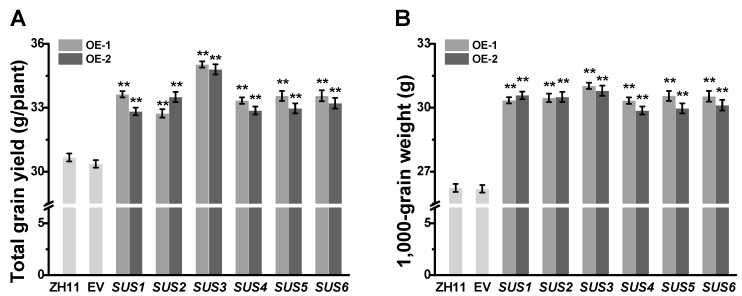
Characterization of grain yield and 1000-grain weight in *OsSUSs*-transgenic rice plants. (**A**) Grain yield of *OsSUS1-6* transgenic plants (*n* = 20). (**B**) Dry weight of 1000 grains. *SUS1*-*SUS6* are the homozygous transgenic rice plants that respectively overexpressed *OsSUS1*-*6* genes in ZH11 background (1000 grains per replicate, *n* = 10 replicates). All data are given as means ± SD. * and ** Indicated significant difference between transgenic line and empty vector transgenic line by Student’s *t*-test at *p* < 0.05 and *p* < 0.01, respectively.

In the field experiments, all transgenic rice lines showed a normal growth and development over the life cycles of rice. Compared with the ZH11 and empty vector (EV) controls, the grain yields per plant of *OsSUSs* transgenic lines were increased by 8–15% (Figure 2A). Statistical analysis showed no significant differences in tiller number per plant, seed number per panicle, and seed setting rate between controls and *OsSUSs* transgenic lines (Table S4). However, 1000-grain weight were significantly increased by 14–18% than that of controls, from 26.24 g and 26.19 g in the ZH11 and EV to 29.87–31.03 g in the transgenic plants (Figure 2B), respectively.

### 2.3. Raised Grain Length in OsSUS3 Transgenic Lines

**Figure 3:**
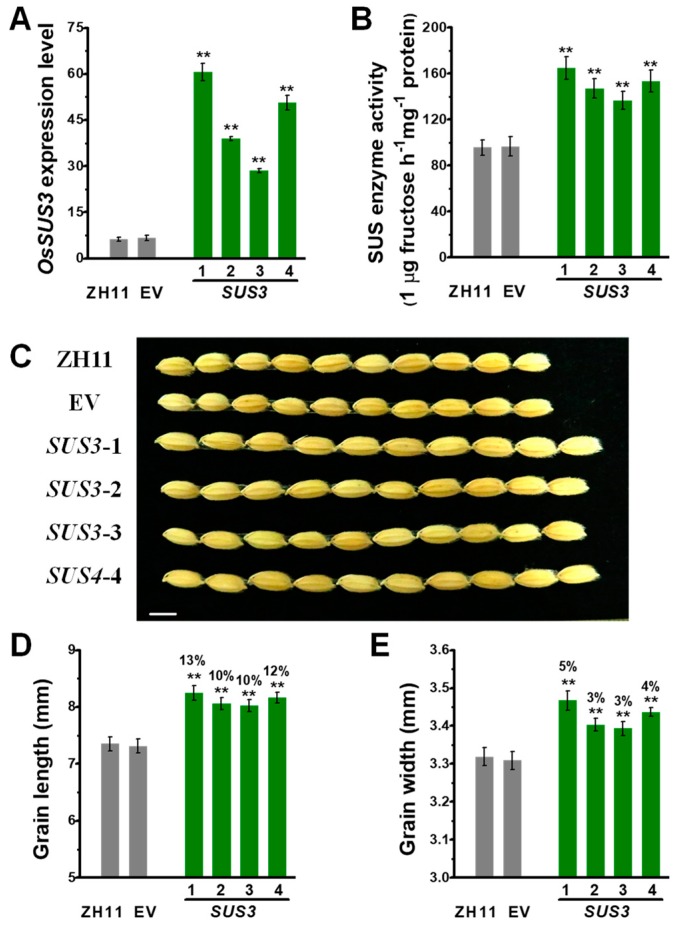
Characterization of grain weight and related traits in *OsSUS3*-transgenic plants. (**A**) Q-PCR analysis of *OsSUS3* expression in four transgenic lines. (**B**) Total OsSUS activity assay. (**C**) Comparison of ZH11, EV and *OsSUS3*-transgenic grains, scale bars as 10 mm. (**D**) Grain length. (**E**) Grain width. All data are given as means ± SD. A Student’s *t*-test performed between transgenic plants and EV as ** *p* < 0.01 and * *p* < 0.05 (*n* = 3 in A, B; *n* = 50 in D, E).

To understand how grain weight are controlled in the *OsSUSs* transgenic rice lines, this study focused to investigate *OsSUS3* transgenic lines for the following experiments, because the OsSUS3 was of much high expression in the spikelet and early endosperm development (Figure 1C) and the *OsSUS3*-OE lines had the largest seeds among all the transgenic lines examined (Figure 2). Based on classic genetic screening, four independent single-locus homozygous transgenic *OsSUS3* lines were obtained, with greatly increased *OsSUS3* expression levels determined by Q-PCR, and significantly elevated SUS enzyme activities by *in vitro* assay (Figure 3A,B).

During the three-year field experiments at different locations (Wuhan and Hainan), all transgenic rice lines showed significantly higher grain yield and 1000-grain weight, compared with those of the ZH11 and EV controls (*p* < 0.05 or *p* < 0.01) (Table 1). As 1000-grain weights were significantly changed, this study examined the grain length, width and thickness in the transgenic rice plants. We observed a substantial increase (10–13%) of grain length, with a slightly increase in grain width (3–5%), and no obvious differences in grain thickness (Figure 3C–E, Figure S2). Hence, the results indicated that the *OsSUS3* overexpression could significantly improve grain weight mainly through increasing grain length and width in the transgenic rice lines. 

### 2.4. Increased Cell Number in OsSUS3-OE Spikelet Hulls

**Figure 4:**
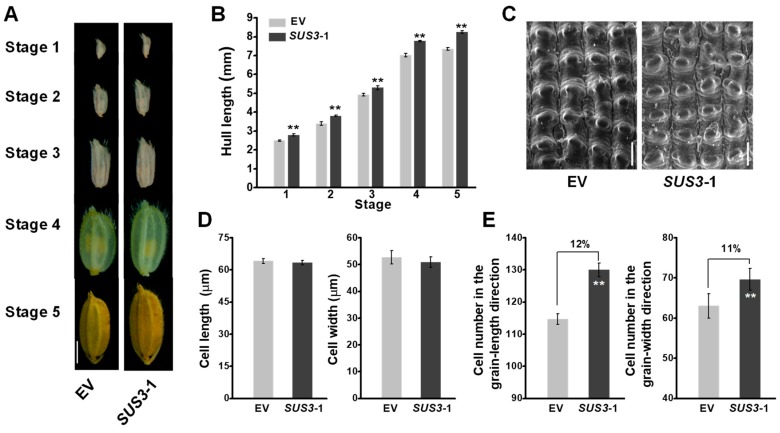
Characterization of hull length and related traits in *OsSUS3*-transgenic plants. (**A**) Light images of hulls at five stages of hull development; scale bars as 2 mm. (**B**) Hull length at five stages of hull development. (**C**) Scanning electron microscope images of glume outer surfaces at mature stage of hull, scale bars as 50 μm (**D**) Cell length of hulls observed in C. (**E**) Cell number of glume outer surfaces. All data are given as means ± SD. A Student’s *t*-test performed between transgenic plants and EV as ** *p* < 0.01 and * *p* < 0.05 (*n* = 50 in B, D, E).

As the spikelet hulls of rice have been proposed to control grain growth for grain size [39], we firstly examined the spikelet hull growth and development. The representative *OsSUS3*-OE line exhibited a consistently increased hull length during the hull development from stage 1 to 5 (Figure 4A,B). Cell proliferation and expansion processes have been known to coordinately regulate spikelet hull growth. To further clarify the causes of the larger grains in *OsSUS3-OE* lines, we examined both cell size and cell number of spikelet hulls. Using scanning electron microscope (SEM), we further observed the outer epidermal cells of spikelet hulls (Figure 4C). As a result, the average length and width of the epidermal cells were not significantly changed in *OsSUS3*-OE line compared with the control (Figure 4D), while the cell numbers both in grain- length and grain- width directions were significantly increased (Figure 4E), suggesting that the larger hull sizes may mainly result from the increased cell numbers. 

### 2.5. Altered Gene Expressions Associated with Cell Division and Cellulose Biosynthesis 

**Figure 5:**
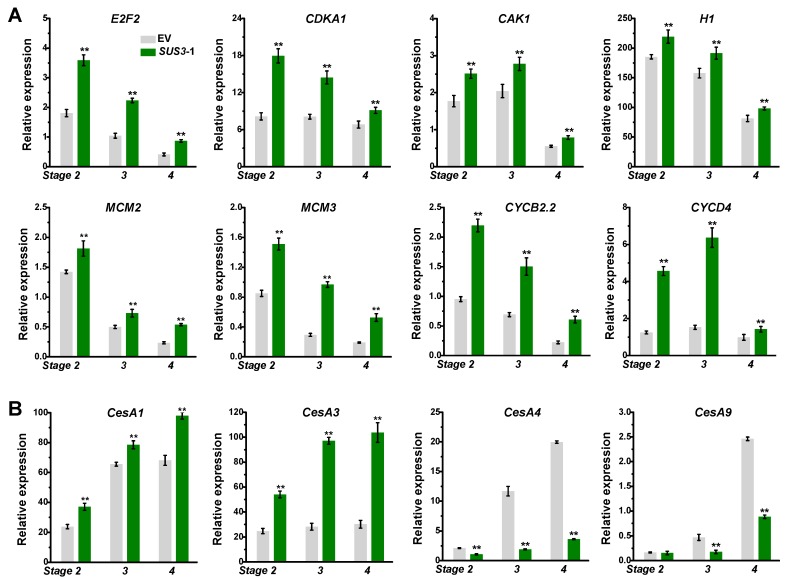
The expression profiling of cell division related genes and cellulose synthase genes. (**A**) Cell division related genes. (**B**) Cellulose synthase genes. All data are given as means ± SD. A Student’s *t*-test performed between transgenic plants and EV as ** *p* < 0.01 and * *p* < 0.05 (*n* = 3).

As plant organ size and cell number is determined by cell division, this study examined the expression of genes related to cell division. Compared to the controls, the relative expression levels of genes involved in cell cycle (*E2F2*, *CDKA1*, *CAK1*, *H1*, *MCM3*, *CYCB2.2*) [8] were considerably up-regulated in *OsSUS3*-OE line during hull development from stages 2 to 4 (Figure 5A), consistent with the increased cell number. Thus, the results indicated that the larger hull sizes in the *OsSUS3* transgenic lines may mainly result from the increased cell numbers in the spikelet hulls by promoting cell division. In addition, the *OsSUS3* transgenic hulls exhibited much higher expressions of *OsCESA1*, *A3* and lower expressions of *OsCESA4*, *A9* genes, which are respectively involved in cellulose biosynthesis of primary and secondary cell walls (Figure 5B). 

### 2.6. Augmented Starch Accumulation in the OsSUS3 Transgenic Grains

**Figure 6:**
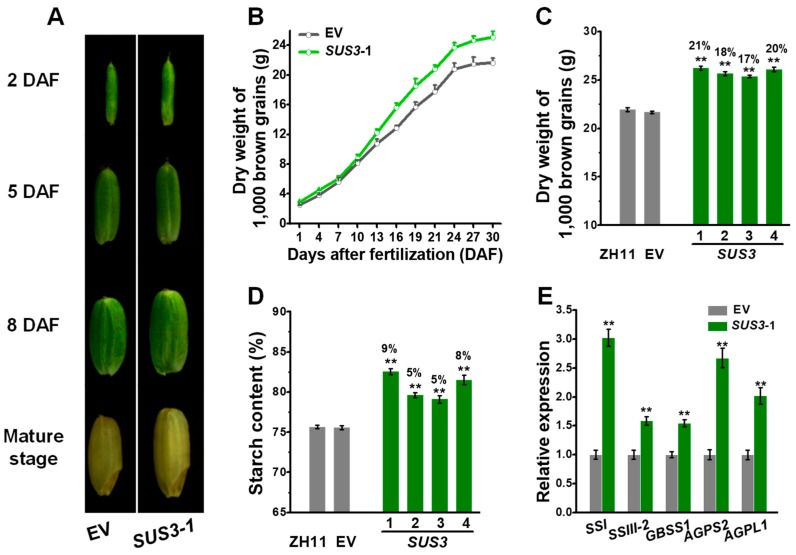
Assessments of grain filling in *OsSUS3*-transgenic plants. (**A**) Images of developing brown grains at four stages (DAF, days after fertilization). (**B**) Time-course of dry weights of 1000 brown grains. (**C**) Dry weight of 1000 brown grains at mature stage. (**D**) Starch content of brown grains at mature stage. (**E**) Q-PCR analysis of starch synthase genes in endosperm. All data are given as means ± SD (*n* = 10 in B, C; *n* = 3 in D, E). A Student’s *t*-test performed between transgenic plants and ZH11 as ** *p* < 0.01 and * *p* < 0.05.

We further compared the sizes of brown grains (grain without hull) between the representative *OsSUS3* transgenic line and the EV control (Figure 6). During grain development, the *OsSUS3* transgenic line showed consistently larger brown grain sizes, resulting in significantly increased 1000 brown grains than those of the EV (Figure 6A,B). In particular, the transgenic lines had increased dry weight of 1000 brown grains by 17–21% at mature stage, compared with the ZH11 and EV (Figure 6C). Because starch is the major component of brown rice grain, we measured that the four *OsSUS3* transgenic lines exhibited higher starch levels by 5–9% than those in the ZH11 (Figure 6D). In terms of the increased starch levels in the transgenic lines, we analyzed the transcript levels of *OsSSI*, *OsSSIII-2*, *OsGBSS1*, *OsAGPS2* and *OsAGPL1* genes, which are preferentially expressed in the endosperm for starch synthesis [40]. In comparison, those genes were much higher expressed in the *OsSUS3* transgenic grains than those in the EV (Figure 6E), consistent with the increased starch contents (Figure 6D) and the enhanced *OsSUS3* expression in the endosperm (Figure S3) of *OsSUS3* transgenic plants. Taken together, the results suggested that the *OsSUS3* overexpression might increase the endosperm volume and up-regulate *OsSSs* and *OsAGPs* expression for starch synthesis, leading to a relatively increased grain weight during grain-filling in the *OsSUS3* transgenic rice plants.

## 3. Discussion

Grain yield in rice is determined by tillers number per plant, grain number per panicle, and grain weight. It is important to increase grain weight for further improvement in grain yield, when tillers number per plant and grain number per panicle reach optimum levels. Grain weight is mainly determined by grain size. In rice, spikelet hulls are proposed to restrict seed growth, thereby determining the final grain size [8,39]. In principle, the spikelet hull growth is determined by cell division, expansion and differentiation [41]. In this study, despite that all *OsSUS-*overexpressed transgenic rice plants showed consistently increased grain yield and 1000-grain weight (Figure 2), an examination from large-scale field experiments is needed in the future. Further analysis in *OsSUS3*-OE plants indicated that the hull size, especially hull length was enhanced (Figure 3). Notably, the *OsSUS3-*OE transgenic rice plants showed consistently increased cell number for large hull size, supported by the enhanced transcript levels of cell division-related genes during hull growth and development (Figure 4 and Figure 5). It has been shown that sucrose metabolism is tightly coupled with sugar signaling by the generation of sugar signaling molecules such as sucrose, glucose, fructose, and trehalose-6-phosphate, and the sugar signaling modulates plant development either directly or through interactions with other signaling pathways, including hormone- and redox-mediated processes [42]. As SUS catalyzes the conversion of sucrose and a nucleoside diphosphate into the nucleoside diphosphate-glucose and fructose, the contents of sugar signaling molecules should be altered in *OsSUSs* overexpressed plants. This may modulates rice hull development by regulating cell division. 

In addition, cell size and shape are basically decided by plant cell walls [43,44,45]. In plants, primary cell wall synthesis is intimately associated with cell division and elongation processes that determine an organ/tissue size, whereas secondary wall synthesis is initiated for the process of cell differentiation and biomass deposition [46,47,48]. As SUS enzymes are characterized to provide UDPG substrates for the biosynthesis of cellulose [25,26,27,28,29,30,33,34], the *OsSUS-*overexpressed transgenic rice plants showed an increased expression of primary cell wall cellulose synthesis genes (*OsCESA1*, *A3*) and the reduced expression of secondary wall cellulose biosynthesis genes (*OsCESA4*, *A9*) during hull growth (Figure 5B). Thus, *OsSUS* overexpression may prolong primary wall cellulose synthesis and consequently delay secondary wall synthesis for enhanced cell division in the hull tissues, resulting in relatively increased cell numbers for larger hull sizes in the transgenic rice plants. 

Grain weight is also determined by the degree of grain filling in crops [5]. Generally, photo-assimilate supply is an important limiting factor for grain filling in the spikelets. The poor grain filling of the spikelets is mainly due to a weak competence for the photo-assimilate, as a result of low grain sink capacity. As SUS has been regarded as a biochemical marker for sink strength [42], the *OsSUS* overexpression may accordingly enhance the sink strength in grain, and increase ADPG substrate for starch biosynthesis, result in increased grain fillings in the transgenic rice plants in this study. In addition, because it has been reported that OsSUS3 provides high-temperature tolerance during the ripening stage in rice [49], it remains interesting to examine whether the abiotic stresses would be enhanced in the *OsSUS-*overexpressed transgenic rice plants in the future studies.

Although six *OsSUSs* family genes exhibit different genomic structures and distinct expression patterns in the life cycle of rice, all selected *OsSUS1-6* overexpressed plants appeared to show increased 1000-grain weight and grain yield in this study. In addition, our preliminary data also showed that the co-overexpression between *OsSUS3* and other five *OsSUSs* genes could not further enhance grain weight (data not shown), suggesting that each OsSUS enzyme may have an identical role in improvements of hull size and grain weight when overexpressed in rice. 

It has been characterized that grain weight is affected by the *GIF1* gene, a cell wall invertase required for carbon partitioning during early grain filling, because the *gif1* mutant shows a slowing down of grain filling for a reduced grain weight [19], whereas the GIF1 overexpressed transgenic maize plants produce larger cobs and kernels for increased grain yield [25]. Since both OsSUS and invertase enzymes could catalyze sucrose version for carbon partitioning regulation, it is assumed that those two enzymes should play a similar role for increased grain weight in the transgenic crops. However, it remains to test which enzyme could be more effective for enhanced grain weight, in particular on grain yield in the field. In addition, it is interesting to explore whether co-overexpression of both SUS and invertase genes could lead to an accumulative enhancement of grain weight in rice and other crops in the future.

## 4. Materials and Methods

### 4.1. Sequence and Phylogenetic Analysis

DNA sequences of *OsSUS* genes were obtained from the rice genome annotation project (RGAP) (http://rice.plantbiology.msu.edu). Exon-intron structure analysis was performed using GSDS (http://gsds. cbi.pku.edu.cn/), and the sequences were aligned using ClustalW program implemented in MEGA7 (https://www.megasoftware.net/). The protein-coding sequences were downloaded from the National Center for Biotechnology Information (NCBI) web site (http://www.ncbi.nlm.nih.gov). One hundred and twelve protein sequences from thirty-seven plant species were collected (Table S1). The phylogenetic tree was constructed by MEGA7 with neighbor-joining (NJ) method. 

### 4.2. Expression Analysis of OsSUS Gene Family

Expression profile data of 33 rice tissue samples (Table S2) in Zhenshan97 (ZS97) were obtained from CREP database (http://crep.ncpgr.cn) and rice transcriptome project using Affymetrix Rice GeneChip microarray [50].

### 4.3. Vectors Construction and Gene Transformation

The full-length cDNA clones of six *OsSUS* were amplified from rice cultivar “Nipponbare” (a *japonica* variety), and inserted into the plant binary vector pCAMBIA1300 (Cambia) driven with maize *polyubiquitin* (Ubi) promoter. The constructs were introduced into *Agrobacterium tumefaciens* strain EHA105 and transferred to rice cultivar “Zhonghua11” (ZH11) by *Agrobacterium*-mediated transformation. The transgenic plants were selected by the PCR analysis using hygromycin gene sequence as primers. The single-locus homozygous *OsSUS1-6* transgenic lines were then identified by the genetic analyses of segregation at 3:1 in the T_1_ generation and no separations in the T_2_ and T_3_ generations (*n* > 30). All primers used for gene cloning were listed in Table S3.

### 4.4. Field Experiments and Plant Sample Collection

Transgenic rice plants were respectively grown in the Experimental Stations of Huazhong Agricultural University, Wuhan and Hainan, China. Conventional rice cropping practices, including irrigation, fertilizer application, and pest control, were applied to the field experiments in this study. The spikelet hulls at five developing stages (stage 1, 2 mm; stage 2, 3 mm; stage 3, 5 mm; stage 4, 7 mm; stage 5, maturity) were collected, frozen in liquid nitrogen and stored at −80 °C until use. The grains of various stages from 1 day after fertilization (DAF) to 30 DAF were collected, dried to constant weight and calculated for 1000-grain weight. Mature grains were used for measuring 1000-grain weight, grain length, width and thickness as previously described by Li et al. [8]. Leica stereomicroscope (Leica S6 D, Leica DFC295 digital camera, Mannheim, Baden-Württemberg, Germany) was used for hull and grain observations. The lengths of hulls at five developing stages were measured using Image J (https://imagej.nih.gov/ij/). For all measurements, grains were obtained from 20 plants grown with 16.7 × 23 cm spacing in paddies under normal cultivation conditions, 1000 seeds per replicate for 1000-grain weight and more than three independent replicates were used for measurements. 

### 4.5. RNA Preparation and Quantitative PCR

Total RNA of plant tissues was extracted using Trizol reagent (Invitrogen, Carlsbad, CA, USA), and reverse-transcribed into cDNA with the GoScript™ Reverse Transcription System (Promega, Madison, WI,, USA). The RT-PCR reaction was performed as described previously [34]. Quantitative PCR reactions were carried out on a Bio-rad MyCycler thermal cycler (Hampton, NH, USA) with the 2 × SYBR Green qPCR Mix (TransGen Co., Ltd., Beijing, China) according to the manufacturer’s instruction. The rice *polyubiquitin* gene (*OsUBQ1*) was used as the internal control. Each measurement was performed using at least two biological samples and each test of sample was conducted with three replicates. The relative quantification of the transcript levels was performed using the comparative *C*t method [34]. All primers used for qPCR were listed in Table S3.

### 4.6. SUS Enzyme Activity Assay

Total proteins were extracted with the buffer (50 mM Hepes-KOH, 10 mM MgCl_2_, 1 mM EDTA, 2 mM DTT, 1 mM PMSF, 5 mM Amino-n-caproic acid, 0.1% *v*/*v* Triton X-100, 10% *v*/*v* glycerol, pH 7.5) [36], and SUS activity was assayed as described [51] with minor modification. Each reaction contained 200 μL enzyme extract and 400 μL reaction buffer with 50 mM Hepes-KOH (pH 6.5), 100 mM sucrose and 2 mM UDP. The control reactions were performed without UDP in the reaction buffer and the produced fructose levels were subtracted. The fructose contents were tested by GC-MS as previously described [52]. Protein concentration was determined by the Bradford method with Bovine Serum Albumin (BSA) as standard, and one unit of enzyme activity is defined as 1 mg enzyme releasing 1 μg fructose h^−1^. Three independent biological replicates were performed for all samples.

### 4.7. Microscope Observation

The glumes outer surfaces of rice spikelet hulls were observed under a scanning electron microscope (SEM) (JSM-6390LV, JEOL, Tokyo, Japan). The spikelet hulls at developing stages were collected, subsequently fixed with 2.5% (*v*/*v*) glutaraldehyde, vacuumed three times, and fixed for at least 24 h. Samples were air-dried, sputter-coated with gold particles, observed and photographed using a scanning electron microscope (JSM-6390LV; JEOL, Tokyo, Japan). The cell density of the glume was calculated as cell number mm^−1^ in longitude from at least 20 replicates [4,13].

### 4.8. Starch Content Assay

The endosperm at different development of *OsSUS3-OE* and wild-type plants were used to measure the content of starch. The starch were collected and assayed by total starch assay kit (Megazyme, Bray, Co. Wicklow, Ireland).

## 5. Conclusions

Individual overexpression of six *OsSUS* genes leads to significantly increased grain weights in all *OsSUS1-6* transgenic rice lines, compared to ZH11 and EV controls. The representative *OsSUS3* transgenic hulls show raised cell numbers for large hull size during hull growth and development by enhancing cell division and related cellulose biosynthesis. Meanwhile, the *OsSUS3* transgenic grains exhibit much starch accumulation for improved grain filling. Hence, this study has demonstrated that OsSUS could enhance cell division in hulls and starch accumulation in grains for increased grain weight in transgenic rice plants.

## Figures and Tables

**Table 1:** Grain-yield related traits of *OsSUS3*-transgenic rice plants in the three-year field experiment.

Year	Transgenic Line		Total Grain Yield (g/Plant)	Percentage of Increased Level (%)	1000-Grain Weight (g)	Percentage of Increased Level (%)	Seeds Per Panicle	Seed Setting Rate (%)	Tillers Per Plant
Year 1	ZH11	ZH11	34.01 ± 1.69		23.78 ± 0.25		133.50 ± 8.94	82.18 ± 2.19	16.83 ± 1.88
	Vector	EV	33.56 ± 2.24		23.63 ± 0.48		136.33 ± 8.53	81.57 ± 1.80	16.67 ± 1.03
	*SU3-*OE	1	37.56 ± 2.83 *	+ 12% ^†^	25.37 ± 0.37 **	+ 7%	125.50 ± 5.09	82.05 ± 2.43	15.67 ± 1.51
		2	36.26 ± 1.47 *	+ 8%	24.77 ± 0.42 **	+ 5%	123.84 ± 7.99	82.21 ± 1.98	16.17 ± 1.17
		3	36.68 ± 2.98	+ 9%	24.85 ± 0.40 *	+ 5%	124.00 ± 8.83	81.49 ± 1.94	16.83 ± 0.75
		4	35.72 ± 1.80 *	+ 6%	25.07 ± 0.27 **	+ 6%	129.33 ± 6.47	83.05 ± 1.53	15.50 ± 1.05
Year 2	ZH11	ZH11	27.82 ± 1.14		22.92 ± 0.68		126.53 ± 4.18	80.56 ± 2.94	13.50 ± 1.05
	Vector	EV	28.44 ± 2.21		22.54 ± 0.87		129.67 ± 6.38	81.50 ± 2.04	14.00 ± 0.89
	*SU3-*OE	1	33.91 ± 0.77 **	+ 19%	24.97 ± 1.30 *	+ 11%	123.84 ± 5.04	83.24 ± 3.31	13.83 ± 0.75
		2	33.90 ± 3.65 **	+ 19%	23.84 ± 0.53 *	+ 6%	127.33 ± 7.23	82.96 ± 3.13	13.33 ± 0.52
		3	31.07 ± 1.27	+ 9%	24.48 ± 1.08 **	+ 9%	120.67 ± 6.68	81.54 ± 2.22	14.00 ± 0.75
		4	31.39 ± 3.03 *	+ 10%	25.93 ± 0.71 **	+ 15%	121.17 ± 5.49	81.54 ± 1.45	13.33 ± 1.03
Year 3	ZH11	ZH11	23.58 ± 0.90		23.77 ± 0.04		123.83 ± 7.65	81.35 ± 2.68	10.20 ± 1.15
	Vector	EV	23.42 ± 1.25		23.76 ± 0.03		127.33 ± 7.76	81.79 ± 1.70	10.50 ± 0.65
	*SU3-*OE	1	26.43 ± 0.86 *	+13%	25.74 ± 0.04 **	+ 8%	121.00 ± 10.00	81.33 ± 2.65	11.50 ± 1.01
		2	25.22 ± 0.33 *	+8%	24.91 ± 0.12 **	+ 5%	129.67 ± 6.35	83.93 ± 0.95	11.20 ± 0.82
		3	25.53 ± 0.87 *	+9%	25.60 ± 0.10 **	+ 8%	128.67 ± 5.47	83.26 ± 1.96	11.40 ± 0.92
		4	26.68 ± 1.60 **	+14%	25.86 ± 0.07 **	+ 9%	127.50 ± 4.42	80.75 ± 3.17	10.50 ± 1.05

* and ** indicated significant difference between transgenic lines and EV by Student’s *t*-test at *p* < 0.05 and *p* < 0.01 (*n* = 20); ^†^ percentage of increased or decreased level between transgenic line and EV by subtraction of two values divided by EV.

## Supplementary Materials

The following are available online at https://www.mdpi.com/1422-0067/20/20/4971/s1.
